# *In vitro* Effect of Dalteparin and Argatroban on Hemostasis in Critically Ill Sepsis Patients with New-Onset Thrombocytopenia

**DOI:** 10.1055/a-2000-6576

**Published:** 2023-01-30

**Authors:** Søren Nygaard, Christine L. Hvas, Anne-Mette Hvas, Kasper Adelborg

**Affiliations:** 1 Department of Clinical Biochemistry, Thrombosis and Hemostasis Research Unit, Aarhus University Hospital, Aarhus, Denmark; 2 Department of Clinical Medicine, Aarhus University, Aarhus, Denmark; 3 Department of Anesthesiology and Intensive Care, Aarhus University Hospital, Aarhus, Denmark; 4 Faculty of Health, Aarhus University, Aarhus, Denmark; 5 Department of Clinical Epidemiology, Aarhus University Hospital, Aarhus, Denmark; 6 Department of Clinical Biochemistry, Gødstrup Regional Hospital, Herning, Denmark

**Keywords:** thrombin, intensive care unit, thromboelastometry, anticoagulant prophylaxis, sepsis, thrombocytopenia

## Abstract

Thrombocytopenia is common among critically ill sepsis patients, while they also hold an increased risk for thromboembolic events. Thus, the choice of anticoagulant prophylaxis for this patient population is challenging. We investigated the *in vitro* effect of low-molecular-weight heparin (dalteparin) and direct thrombin inhibitor (argatroban) on the hemostasis in blood from sepsis patients with new-onset thrombocytopenia. Thrombocytopenia was defined as a platelet count drop of ≥30% and/or from >100 × 10 ^9^ /L to 30 to 100 × 10 ^9^ /L within 24 hours prior to inclusion. We included five healthy individuals and ten patients. Analyses of thrombin generation (Calibrated Automated Thrombogram), thrombin-antithrombin (TAT) complex levels, prothrombin fragment 1+2 (F1+2), and rotational thromboelastometry (ROTEM) were performed. Based on dose–response relationships investigated in healthy blood, patient samples were spiked with prophylactic (0.25 IU/mL) and therapeutic (0.75 IU/mL) dalteparin and low (0.25 µg/mL) and high (0.50 µg/mL) argatroban concentrations, each with a sample without anticoagulant. In patients, the endogenous thrombin potential was markedly lower in therapeutic dalteparin samples than in samples without anticoagulant [median (range): 29 (0–388) vs. 795 (98–2121) nM × min]. In high argatroban concentration samples, thrombin lag time was longer than in samples without anticoagulant [median (range): 15.5 (10.5–20.2) versus 5.3 (2.8–7.3) min]. Dalteparin and argatroban both increased clotting time but did not affect maximum clot firmness in the ROTEM INTEM assay. Six patients had elevated TAT and eight patients had elevated F1 + 2. In conclusion, dalteparin mainly affected the amount of thrombin generated and argatroban delayed clot initiation in critically ill sepsis patients with new-onset thrombocytopenia. Neither anticoagulant affected clot strength.

## Introduction

Coagulation abnormalities are common in patients with sepsis. 1 The clinical presentation can vary from mild thrombocytopenia to overt disseminated intravascular coagulation (DIC) with thrombosis, organ dysfunction, and death. 2 3 Patients with sepsis who develop thrombocytopenia have a higher risk of major bleeding and acute kidney injury, longer stay at the intensive care unit (ICU), and higher mortality than patients with normal platelet counts. 4 Furthermore, a large decline in platelet count predicts a poor outcome, regardless of the absolute platelet count. 5 Although the pathophysiology of thrombosis formation is complex and may include multiple pathways, inhibition of excessive thrombin generation at an early stage of sepsis-induced consumption of platelets could potentially prevent the widespread development of microthrombi, but firm evidence on the choice of anticoagulant prophylaxis in these patients is lacking. Theoretically, a direct thrombin inhibitor as an early anticoagulant intervention could be of interest for this purpose. 6 The direct thrombin inhibitor argatroban has a short half-life (40–50 min.), is predominantly eliminated via hepatic metabolism, and unlike low-molecular-weight heparin (LMWH), *e.g.* , dalteparin, it is not dependent on antithrombin for its anticoagulant activity. 7 Argatroban has been approved as an alternative anticoagulant in patients with heparin-induced thrombocytopenia, and when using dosage adjustments, it is considered safe in critically ill patients. 8 9 10 The role of heparin as an anticoagulant treatment in sepsis remains controversial but may reduce mortality. 11

The aim of this study was to investigate the *in vitro* effect of dalteparin and argatroban on thrombin generation and global dynamic hemostasis in blood samples from critically ill sepsis patients with new-onset thrombocytopenia.

## Materials and Methods

### Study Design

The study was a cross-sectional *in vitro* study performed at the Department of Clinical Biochemistry and the Department of Anesthesiology and Intensive Care at Aarhus University Hospital, Aarhus, Denmark. The Danish Data Protection Agency (case no.: 1-16-02-382-21) and The Central Denmark Region Committees on Health Research Ethics (case no.: 1-10-72-320-21) approved the study. The study followed the Declaration of Helsinki, and all participating patients or a surrogate gave informed consent to inclusion.

### Study Populations

First, we used blood samples from five healthy individuals that did not receive any medication (three males and two females, aged 30–51 years) to investigate the effects of dalteparin and argatroban on hemostasis. Second, we included ten patients (≥18 years of age) with sepsis and new-onset thrombocytopenia at the ICU. Sepsis was defined as a known or suspected infection based on cultures and clinical assessment by physicians and a ≥2 points increase in Sequential Organ Failure Assessment (SOFA) score compared with the SOFA score upon admission to the ICU. 12 New-onset thrombocytopenia was defined as a drop in platelet count of ≥30% and/or from >100 × 10 ^9^ /L to 30 to 100 × 10 ^9^ /L within 24 hours prior to inclusion. A ≥2 points increase in SOFA score solely based on platelet count was not considered valid for inclusion. Patients were excluded from the study if they fulfilled one or more of the following criteria: (1) Therapeutic anticoagulant treatment with unfractionated heparin, LMWH, vitamin K antagonists, or direct oral anticoagulants, and/or dual antiplatelet treatment, (2) active, major bleeding according to the International Society on Thrombosis and Haemostasis (ISTH) criteria, 13 (3) mechanical circulatory support systems, (4) platelet count <30 × 10 ^9^ /L, and (5) presence of any disorder other than sepsis that was more likely to explain the drop in platelet count.

### Blood Sampling and Preparation

From a non-heparinized arterial cannula, blood samples were collected in 3.5 mL citrated tubes (Vacuette, 3.5 mL, sodium citrate 3.2%), 1.8 mL citrated tubes (BD Vacutainer, 1.8 mL, 0.109 M sodium citrate), and EDTA tubes (BD Vacutainer, 3.0 mL, K2 ethylene diamine tetraacetic acid 5.4 mg). The first tube was discarded. If a patient received prophylactic LMWH, blood sampling was done at least 12 hours after the last injection.

Immediately after blood sampling, 1.8 mL citrated tubes and EDTA tubes were analyzed for coagulation markers without any addition of anticoagulants: antifactor Xa (anti-Xa), international normalized ratio (INR), activated partial thromboplastin time (aPTT), thrombin time, fibrinogen, fibrin D-dimer, and antithrombin; and platelet markers: platelet count, immature platelet fraction, immature platelet count, and mean platelet volume (details about these analyses are provided below). Within 3 hours, the 3.5 mL citrated blood samples were analyzed using rotational thromboelastometry (ROTEM). Within 1 hour, blood was centrifuged for 25 minutes (3,345 g at 20°C) for later measurement of thrombin generation, thrombin-antithrombin (TAT) complex levels, prothrombin fragment 1 + 2 (F1 + 2) concentration, anti-Xa, and aPTT. Plasma for thrombin generation was centrifuged again for 15 minutes (2,500 g at 20°C) to obtain platelet-poor plasma (PPP). All plasma samples were stored at −80°C until analyzed. Apart from TAT and F1 + 2 samples, dalteparin and argatroban were added to citrated blood samples used for ROTEM, thrombin generation, anti-Xa, and aPTT to evaluate their anticoagulant effect.

### Laboratory Tests

#### Thrombin Generation Assay

Thrombin generation was evaluated by the Calibrated Automated Thrombogram (CAT) (Thrombinoscope BV, Maastricht, the Netherlands). Before analysis, frozen PPP samples were thawed for 5 minutes in a water bath at 37°C followed by ultracentrifugation for 3 minutes (16,000 g at 20°C). All PPP samples were measured in duplicate by the Thrombinoscope software (Thrombinoscope BV) for 60 minutes. In a 96-well micro titer plate, a fluorescent peptide substrate is catalyzed by thrombin to release a fluorophore. The fluorescent signal is then measured using the Fluoroskan Ascent plate reader (Thermo Fisher Scientific, Helsinki, Finland). The CAT reagents (Thrombinoscope BV) include the trigger PPP-reagent (5 pM tissue factor and 4 µM phospholipids), Thrombin Calibrator, and FluCa-kit (mixture of Fluo-Substrate and Fluo-Buffer). Thrombin Calibrator was added to wells containing PPP samples without anticoagulant. The measurements included lag time (min), peak thrombin (nM), time-to-peak (min), and area under the curve termed as the endogenous thrombin potential (ETP, nM × min). Peak thrombin values ≤5 nM were interpreted as no thrombin generation.

#### Rotational Thromboelastometry

Blood samples for ROTEM (Instrumentation Laboratory, Bedford, United States) were left to rest for 30 minutes before analysis, and during the last 10 minutes of rest, they were incubated at 37°C. We used the four standard ROTEM assays: EXTEM, INTEM, FIBTEM, and HEPTEM. Analysis was performed according to the manufacturer's instructions. The following parameters were recorded: clotting time (CT, s), maximum velocity (MaxVel, mm/min), time to maximum velocity (MaxVel-t, s), and maximum clot firmness (MCF, mm).

#### Thrombin-Antithrombin and Prothrombin Fragment 1 + 2 Assays

Using commercial enzyme-linked immunosorbent assays, we measured TAT complex levels (Enzygnost TAT Micro, Siemens Healthcare GmbH, Erlangen, Germany) and F1 + 2 concentration (Enzygnost F1 + 2 Mono, Siemens Healthcare GmbH) in duplicate. Samples with a coefficient of variance above 10% were repeated.

#### Coagulation and Platelet Assays

Coagulation analyses were all measured on Sysmex CS-5100 System (Siemens Healthcare GmbH, Erlangen, Germany): Anti-Xa (BIOPHEN Heparin LRT reagent, without antithrombin addition), INR (Medirox Owren's PT reagent), aPTT (Siemens Dade Actin FS reagent), thrombin time (Siemens test thrombin reagent), fibrinogen (functional, Clauss, Siemens Dade thrombin reagent), fibrin D-dimer (immunoturbidimetric method, Siemens INNOVANCE reagent), and antithrombin (Siemens INNOVANCE reagent). Platelet markers were measured on Sysmex XN-9000 (Sysmex, Kobe, Japan): Platelet count, mean platelet volume, immature platelet count, and immature platelet fraction.

### Spiking of Blood Samples

For spiking of blood samples, we used dalteparin (Fragmin, Pfizer ApS, Ballerup, Denmark) and argatroban (Novastan, Mitsubishi Tanabe Pharma GmbH, Düsseldorf, Germany) diluted in 20 mM HEPES buffered saline (150 mM sodium chloride, pH 7.4). Samples without anticoagulant were used as controls, and they were made by adding HEPES buffer to blood from each patient to account for the dilution effect when adding dalteparin and argatroban.

We performed titrations to final plasma concentrations of 0.00 to 1.00 IU/mL for dalteparin and 0.00 to 1.50 µg/mL for argatroban to establish dose–response relationships in blood from healthy individuals and to define estimated clinically relevant drug concentrations. We assumed that plasma accounted for 55% of the blood volume. The spiked blood samples were evaluated by thrombin generation, ROTEM, anti-Xa, and aPTT. The target anti-Xa plasma range of dalteparin administered subcutaneously has been suggested to be 0.20 to 0.50 IU/mL for prophylactic dosage 14 and 0.50 to 1.00 IU/mL for therapeutic dosage. 15 According to the manufacturer, argatroban is monitored by using aPTT aiming between 1.5 and 3 times the baseline aPTT, not exceeding 100 seconds. 16 Based on these plasma ranges and dose-response relationships in healthy blood, patient blood samples were spiked to undergo five separate analyses: (1) HEPES buffer alone (sample without anticoagulant), (2) 0.25 IU/mL dalteparin (prophylactic dose), (3) 0.75 IU/mL dalteparin (therapeutic dose), (4) 0.25 µg/mL argatroban (low concentration), and (5) 0.50 µg/mL argatroban (high concentration).

### Reference Intervals

Published data by our research group from 73 healthy individuals (age 20–60) in ROTEM 17 and from 124 healthy individuals (age 21–66) in the TAT and F1 + 2 assays 18 were used for reference intervals. All data were equally gender distributed.

### Medical Record Data Collection

Data for patient characteristics were collected from electronic medical records and included: age, sex, body mass index (BMI), the Simplified Acute Physiology Score-III (SAPS III), 19 comorbidity (including hypertension, hypercholesterolemia, type 2 diabetes, and the Charlson comorbidity index 20 ), infection (including septic shock and cultures), biochemical characteristics (including platelet, coagulation, organ, and infection markers), and treatment/procedures (including intubation, dialysis, and transfusions). Thirty-day all-cause mortality was recorded.

Based on coagulation and organ markers, disease scores were calculated and included the SOFA score, 12 ISTH overt DIC score, 21 22 23 the Japanese Association for Acute Medicine (JAAM) 2016 DIC score, 24 and the sepsis-induced coagulopathy (SIC) score. 25 We changed the cut-off values for D-dimer and antithrombin in the DIC scoring systems to be consistent with the reference intervals in healthy Danish adults. 22

### Data Management and Representation

Data management of the study population was achieved using the research electronic data capture (REDCap, Vanderbilt University, United States) tools hosted at Aarhus University, Denmark. 26 No formal sample size calculation was performed because this study was purely exploratory. Analysis was made to divide patients into two groups based on whether peak thrombin values were ≥100 nM or <100 nM. All data are presented as medians with range. Statistics and graphs were made in GraphPad Prism version 9.4.0 (GraphPad Software, San Diego, California, United States).

## Results

### Patient Characteristics

Patient characteristics are provided in Table 1 . At inclusion, eight patients had moderate thrombocytopenia with platelet counts 50 to 100 × 10 ^9^ /L and two patients had severe thrombocytopenia with platelet counts 30 to 50 × 10 ^9^ /L. Apart from sepsis assessment by a physician, six patients had a positive blood culture within 1 week from inclusion in the study. The SOFA score had a median (range) of 12 (5–17), and after removing the platelet component, the SOFA score had a median (range) of 10 (3–15).

**Table 1:** Demographic, clinical, and biochemical characteristics of the study population ( *n* = 10)

**Demography**	
Age, years	70.5 (48–85)
Female, *n* (%)	5 (50%)
BMI, kg/m ^2^	29.3 (15–43.9)
SAPS III	58 (45–84) a
**Comorbidity**	
Hypertension, *n* (%)	8 (80%)
Hypercholesterolemia, *n* (%)	6 (60%)
Diabetes mellitus type 2, *n* (%)	5 (50%)
Charlson comorbidity index	1.5 (0–8)
**Infection**	
Septic shock within 24 hours prior to inclusion, *n* (%)	8 (80%)
Positive blood culture within 1 wk, *n* (%)	6 (60%)
Bacteria in one or more cultures within 1 wk, *n* (%)	8 (80%)
**Biochemical characteristics (reference interval)**	
*Platelet markers*	
Platelet count, x10 ^9^ /L (145–400)	74 (41–99)
Absolute decrease in platelets within 24 hours prior to inclusion, x10 ^9^ /L	44 (23–132)
Relative decrease in platelets within 24 hours prior to inclusion, %	45 (19–62)
Immature platelet count, x10 ^9^ /L (4.4–26.7)	9.2 (4.9–15.5)
Immature platelet fraction (0.016–0.126)	0.137 (0.086–0.201)
Mean platelet volume, fl (6.5–11.0)	12.4 (11.2–12.7) b
*Coagulation markers*	
INR (<1.2)	1.4 (1.1–2.0)
aPTT, s (0–29)	31 (22–62)
Fibrinogen, µmol/L (5.5–12)	15.6 (2.5–26.4)
D-dimer, mg/L (<0.8)	8.6 (3.9–20) c
Antithrombin, IU/L (0.8–1.2)	0.58 (0.41–0.71)
*Organ markers*	
Hemoglobin, mmol/L (7.3–10.5)	7.4 (6.2–8.7)
Arterial lactate, mmol/L (0.5–2.5)	2.4 (0.5–4.8)
Creatinine, µmol/L (45–105)	151 (53–276)
Bilirubin, µmol/L (5–25)	17 (6–131)
*Infection markers*	
Leucocytes, x10 ^9^ /L (3.5–10)	13.5 (5.8–50.5)
CRP, mg/L (<8)	264 (87–398)
**Treatment**	
Intubation, *n* (%)	4 (40%)
Dialysis, *n* (%)	1 (10%)
Transfusions within 24 h prior to inclusion, *n* (%)	1 (10%)
Red blood cells, *n* (%)	0 (0%)
Fresh frozen plasma, *n* (%)	1 (10%)
Platelets, *n* (%)	0 (0%)
**Disease scores**	
SOFA score	12 (5–17)
ISTH overt DIC score	5 (3–8)
JAAM DIC score	5 (2–8)
SIC score	6 (4–6)
**Mortality**	
30-day mortality, *n* (%)	3 (30%)

Abbreviations: aPTT, activated partial thromboplastin time; CRP, C-reactive protein; INR, international normalized ratio; ISTH, International Society on Thrombosis and Haemostasis; JAAM, Japanese Association for Acute Medicine; SAPS, Simplified Acute Physiology Score; SIC, sepsis-induced coagulopathy; SOFA, Sequential Organ Failure Assessment.

Note: Data are shown as numbers (%) or medians (range). The values are at inclusion unless stated otherwise. Reference intervals are for Danish adults. Reference intervals for platelets, hemoglobin, and creatinine have been merged for males and females.

a One missing value.

b Two missing values.

c D-dimer >20.0 mg/L was assigned the value 20.0 mg/L.

All patients had a fibrin D-dimer value above the upper reference value of 0.80 mg/L and antithrombin levels below the lower reference value of 0.80 IU/L. Six patients had TAT complex levels above the upper reference value of 13.0 µg/L with a median (range) of 14.7 µg/L (8.9–119.2), and eight patients had F1 + 2 concentrations above the upper reference value of 320 pM with a median (range) of 673 pM (166–2,627). Five patients both had elevated TAT and F1 + 2. Seven patients had DIC according to the JAAM DIC score and five of these seven patients also had an ISTH DIC score consistent with DIC. Three patients died within 30 days at the ICU.

### Spiking Samples from Healthy Individuals and Patients with Dalteparin

**Fig. 1:**
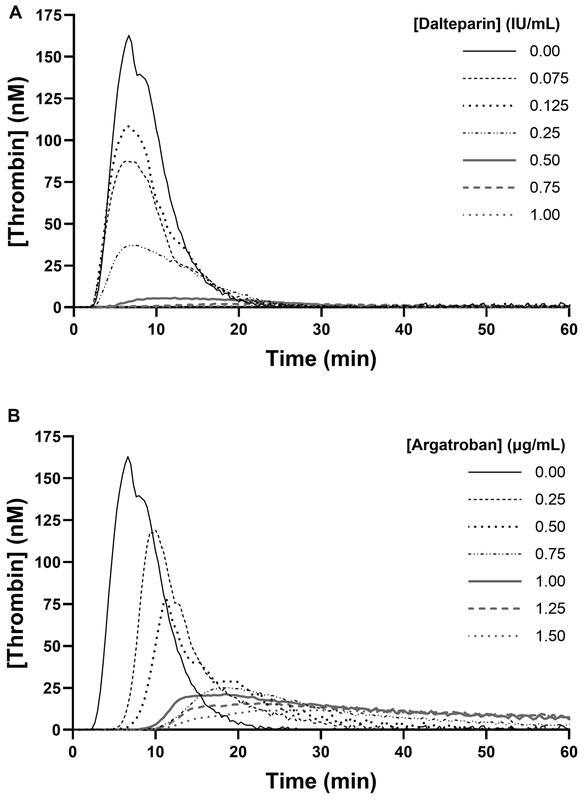
Effect of increasing plasma concentrations of **(A)** dalteparin (0.00 to 1.00 IU/mL) and **(B)** argatroban (0.00 to 1.50 µg/mL) on thrombin generation in platelet-poor plasma samples from healthy individuals ( *n* = 5) triggered with 5 pM tissue factor and 4 µM phospholipids. Data are shown as median curves.

In plasma from healthy individuals, addition of dalteparin (0.00 to 1.00 IU/mL) decreased peak thrombin and ETP in a dose-dependent manner ( Table 2 and Fig. 1A ). No substantial increase was found in lag time and time-to-peak until concentrations at 0.5 IU/mL and above, suggesting no thrombin generation ( Fig. 1A ). Individual curves for dalteparin (0.00 to 1.00 IU/mL) in samples from healthy individuals are provided in the supplementary material ( Fig. S1 ). Dalteparin dose-dependently increased anti-Xa, INTEM-CT, and INTEM-MaxVel-t, whereas INTEM-MaxVel and INTEM-MCF were unaffected ( Table 2 ). Dalteparin had no effect on EXTEM, and therefore these results are not shown for healthy individuals and patients. Likewise, FIBTEM was not affected, and when adding heparinase in HEPTEM, the effect of dalteparin was neutralized.

**Table 2:** Effect of increasing plasma concentrations of dalteparin (0.00 to 1.00 IU/mL) and argatroban (0.00 to 1.50 µg/mL) on anti-factor Xa (anti-Xa), activated partial thromboplastin time (aPTT), INTEM parameters, and thrombin generation parameters in healthy individuals ( *n* = 5)

[Dalteparin], IU/mL	0.00	0.075	0.125	0.25	0.50	0.75	1.00
Anti-Xa, IU/mL	0.00	0.19 (0.16–0.24)	0.20 (0.17–0.22)	0.34 (0.31–0.36)	0.60 (0.56–0.67)	0.89 (0.78–0.94)	1.17 (1.01–1.19)
INTEM parameters							
CT, s	204 (157–218)	227 (203–247)	223 (207–239)	249 (218–259)	274 (228–286)	306 (257–339)	352 (261–368)
MaxVel, mm/s	16 (14–23)	15 (14–22)	16 (14–21)	16 (13–19)	15 (13–20)	13 (12–18)	12 (10–19)
MaxVel-t, s	229 (181–250)	254 (229–290)	263 (233–272)	284 (250–293)	315 (260–325)	343 (293–382)	397 (288–415)
MCF, mm	57 (55–65)	57 (56–65)	59 (55–66)	58 (57–66)	58 (55–66)	56 (55–64)	58 (53–67)
Thrombin generation parameters							
Lag time, min	3.3 (2.3–3.3)	2.7 (2.3–3.3)	3.0 (2.7–3.3)	3.3 (3.0–4.0)	5.0 (4.0–5.8)	6.7 (5.8–7.3)	9.7 (8.2–13.3)
Peak thrombin, nM	165 (127–185)	88 (36–182)	108 (63–153)	37 (21–76)	5.4 (4.4–20)	2.1 (1.7–5.2)	1.1 (0.9–1.8)
Time-to-peak, min	7.0 (5.7–7.7)	6.7 (5.7–8.0)	6.7 (6.0–8.0)	7.3 (7.0–9.2)	11.2 (8.5–13.2)	24.0 (12.0–29.3)	42.0 (25.8–46.5)
ETP, nM × min	1,202 (1,094–1,324)	832 (446–1143)	950 (670–1058)	457 (297–706)	114 (91–265)	70 (60–123)	47 (37–68)
**[Argatroban], µg/mL**	**0.00**	**0.25**	**0.50**	**0.75**	**1.00**	**1.25**	**1.50**
aPTT, s	25 (22–27)	38 (32–40)	42 (41–49)	50 (46–56)	53 (47–59)	58 (51–62)	65 (54–68)
aPTT ratio	1.00	1.48 (1.45–1.54)	1.70 (1.64–1.91)	1.93 (1.88–2.24)	2.12 (2.00–2.27)	2.31 (2.16–2.32)	2.45 (2.32–2.72)
INTEM parameters							
CT, s	208 (180–220)	312 (270–316)	337 (323–353)	359 (324–384)	369 (350–412)	404 (385–429)	429 (412–432)
MaxVel, mm/s	17 (13–24)	15 (13–22)	16 (14–19)	14 (13–19)	15 (13–18)	15 (12–16)	14 (13–16)
MaxVel-t, s	238 (205–253)	358 (298–358)	385 (361–409)	415 (361–437)	418 (409–476)	455 (427–495)	474 (451–502)
MCF, mm	58 (56–67)	57 (56–66)	60 (57–68)	60 (55–68)	60 (57–67)	59 (57–65)	59 (56–66)
Thrombin generation parameters							
Lag time, min	3.2 (2.7–3.3)	6.7 (5.7–8.3)	8.5 (7.0–11.2)	10.5 (8.3–11.7)	10.7 (9.0–14.0)	11.7 (10.2–13.5)	12.5 (10.8–15.7)
Peak thrombin, nM	165 (123–182)	125 (76–136)	89 (29–95)	26 (15–59)	21 (15–41)	16 (13–27)	12 (8.8–22)
Time-to-peak, min	7.0 (6.0–8.0)	9.7 (8.7–12.8)	12.7 (10.2–19.2)	17.8 (12.7–20.2)	16.8 (15.2–27.3)	20.2 (18.5–24.0)	27.0 (21.2–32.0)
ETP, nM × min	1,213 (1,059–1,369)	953 (837–1,061)	737 (654–901)	615 (425–803)	681 (517–710)	565 (387–751)	441 (385–683)

Abbreviations: CT, clotting time; ETP, endogenous thrombin potential; IU, international units; MaxVel, maximum velocity; MaxVel-t, time to maximum velocity; MCF, maximum clot firmness.

Note: Data are shown as medians (range). Ratios are calculated based on the samples without anticoagulant.

**Fig. 2.**
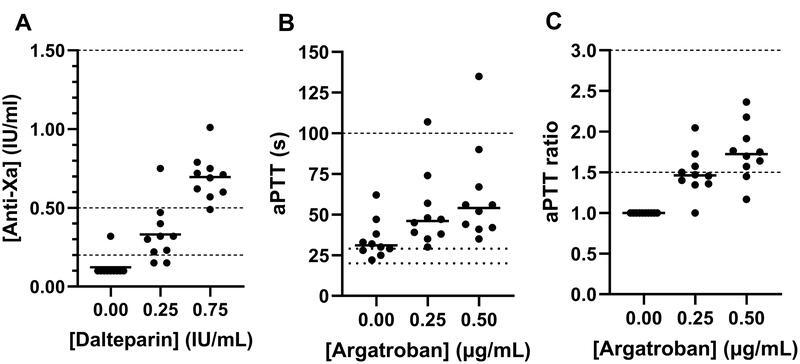
Coagulation assays in critically ill sepsis patients with new-onset thrombocytopenia ( *n* = 10) consisting of: **(A)** Anti-factor Xa (Anti-Xa) in samples without anticoagulant (0.00) and in 0.25 IU/mL and 0.75 IU/mL dalteparin samples. **(B)** Activated partial thromboplastin time (aPTT) and **(C)** aPTT ratios in samples without anticoagulant (0.0) and in 0.25 µg/mL and 0.50 µg/mL argatroban samples. Solid lines are group medians, dotted lines are reference intervals for healthy Danish adults, and dashed lines are plasma concentration ranges following the guidelines when treating patients with dalteparin and argatroban.

Nine patients had the lowest measurable anti-Xa levels of <0.10 IU/mL at inclusion, whereas one patient had a value of 0.32 IU/mL ( Fig. 2A ). In 0.25 IU/mL dalteparin samples, seven patients had anti-Xa levels within the 0.20 to 0.50 IU/mL prophylactic range, two were below this range, and one was above. In 0.75 IU/mL dalteparin samples, eight patients were within the 0.50 to 1.00 IU/mL therapeutic range, one was just below at 0.49 IU/mL, and one just above at 1.01 IU/mL.

**Fig. 3.**
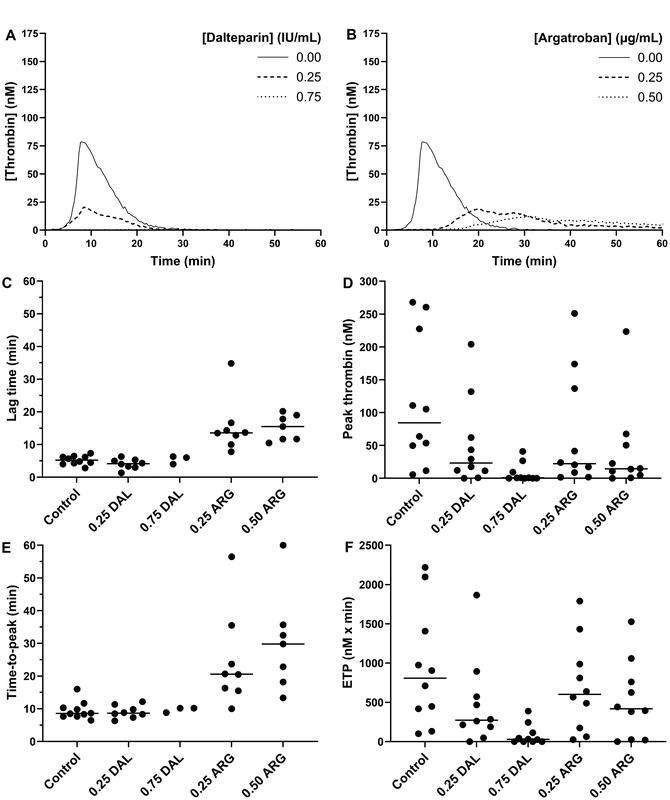
Thrombin generation in platelet-poor plasma samples without anticoagulant (0.00), 0.25 IU/mL and 0.75 IU/mL dalteparin (DAL) samples, and 0.25 µg/mL and 0.50 µg/mL argatroban (ARG) samples from critically ill sepsis patients with new-onset thrombocytopenia ( *n* = 10). Plasma was triggered with 5 pM tissue factor and 4 µM phospholipids. Thrombin generation curves for dalteparin **(A)** and argatroban **(B)** are shown as medians. Lag time **(C)** , peak thrombin **(D)** , time-to-peak **(E)** , and ETP **(F)** are shown as individual values with group medians as solid lines. Group medians for 0.75 IU/mL dalteparin samples in (C) and (E) could not be calculated due to no thrombin generation in seven samples.

Dalteparin affected peak thrombin and ETP, while lag time and time-to-peak did not change notably ( Fig. 3C – F ). Compared with samples without anticoagulant, 0.25 IU/mL and 0.75 IU/mL dalteparin caused a decrease in peak thrombin (84.5 [5.7–267.9] vs. 23.4 [0–204.0] and 0.7 [0–40.9] nM) and a decrease in ETP (808 [101–2,219] vs. 273 [0–1,865] and 29 [0–388] nM × min). Two 0.25 IU/mL and seven 0.75 IU/mL dalteparin samples demonstrated no thrombin generation with peak thrombin values ≤5 nM ( Fig. 3D ). Because most samples with 0.75 IU/mL dalteparin resulted in no thrombin generation, it was not possible to calculate a group median for lag time and time-to-peak ( Fig. 3C,E ). However, compared with samples without anticoagulant, lag time did not increase in 0.25 IU/mL dalteparin samples (5.3 [2.8–7.3] vs. 4.2 [1.3–6.3] min) nor did time-to-peak (8.6 [6.5–16.0] vs. 8.7 [6.3–12.2] min).

**Fig. 4.**
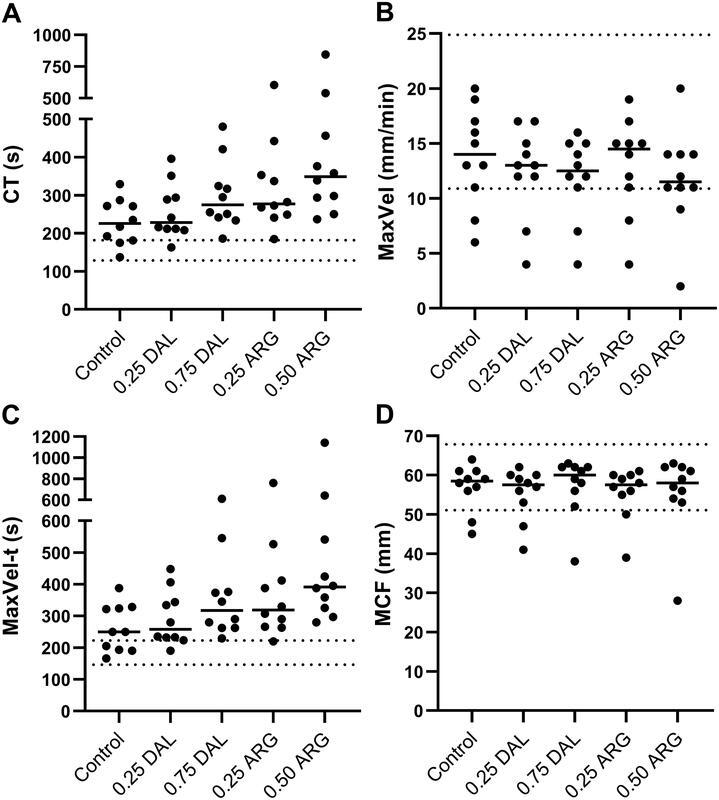
( **A** ) Clotting time (CT), ( **B** ) maximum velocity (MaxVel), ( **C** ) time to maximum velocity (MaxVel-t), and ( **D** ) maximum clot firmness (MCF) evaluated by INTEM in whole blood samples without anticoagulant (Control), 0.25 IU/mL and 0.75 IU/mL dalteparin samples (DAL), and 0.25 µg/mL and 0.50 µg/mL argatroban samples (ARG) from critically ill sepsis patients with new-onset thrombocytopenia ( *n* = 10). Data are shown as individual values. Solid lines are group medians and dotted lines are reference intervals.

Fig. 4 shows that, compared with samples without anticoagulant, 0.75 IU/mL dalteparin prolonged INTEM-CT (226 [137–329] vs. 275 [186–480] s), and likewise, INTEM-MaxVel-t was prolonged (250 [166–388] vs. 318 [229–610] s). Samples with 0.25 IU/mL dalteparin did not affect INTEM-CT nor INTEM-MaxVel-t. Neither concentration of dalteparin affected MaxVel and MCF, apart from one patient sample.

### Spiking Samples from Healthy Individuals and Patients with Argatroban

In plasma from healthy individuals, addition of argatroban (0.00 to 1.50 µg/mL) demonstrated a dose-dependent increase in lag time and time-to-peak and a dose-dependent decrease in peak thrombin when added to plasma from healthy individuals ( Table 2 and Fig. 1B ). Argatroban also decreased ETP, but in samples containing 0.75 to 1.5 µg/mL argatroban, thrombin generation did not cease during the 60-minute runtime thereby only moderately decreasing ETP. Individual curves for argatroban (0.00 to 1.50 µg/mL) in samples from healthy individuals are provided in the supplementary material ( Fig. S2 ). Furthermore, argatroban caused a dose-dependent increase in aPTT, INTEM-CT, and INTEM-MaxVel-t, but neither INTEM-MaxVel nor INTEM-MCF were influenced ( Table 2 ). Argatroban also increased CT in EXTEM, FIBTEM, and HEPTEM.

Samples without anticoagulant from six patients had aPTT values above the reference interval ( Fig. 2B ). In 0.25 µg/mL argatroban samples, aPTT varied from no change to excessive aPTT prolongation >100 seconds ( Fig. 2B ). Four and eight patients had an aPTT ratio between 1.5 and 3 times the baseline aPTT in 0.25 µg/mL and 0.50 µg/mL argatroban samples, respectively ( Fig. 2C ).

When spiking patient plasma samples with argatroban, this caused a prolongation in lag time and time-to-peak and a decrease in peak thrombin and ETP ( Fig. 3C – F ). Compared with samples without anticoagulant, both 0.25 µg/mL and 0.50 µg/mL argatroban increased lag time (5.3 [2.8–7.3] vs. 13.6 [7.8–34.8] and 15.5 [10.5–20.2] min), increased time-to-peak (8.6 [6.5–16.0] vs. 20.6 [10.0–56.5] and 29.8 [13.3–60.0] min), decreased peak thrombin (84.5 [5.7–267.9] vs. 22.3 [0–251.0] and 14.5 [0–223.3] nM), and decreased ETP (808 [101–2,219] vs. 603 [26–1,789] and 419 [0–1,527] nM × min). ETP was not inhibited as much as peak thrombin, partly because some thrombin generation curves did not cease during the 60-minute runtime ( Fig. 3D,F ). Two 0.25 µg/mL and three 0.50 µg/mL argatroban samples demonstrated no thrombin generation with peak thrombin values ≤5 nM ( Fig. 3D ). Individual curves for dalteparin and argatroban in patient samples are provided in the supplementary material ( Fig. S3 ).

As shown in Fig. 4 , compared with patient samples without anticoagulant, INTEM-CT was prolonged in 0.25 µg/mL and 0.50 µg/mL argatroban samples (226 [137–329] vs. 278 [185–603] and 349 [237–845] s), and the same pattern was evident for INTEM-MaxVel-t (250 [166–388] vs. 319 [220–761] and 392 [280–1,142] s). Argatroban did not affect MaxVel and MCF, except for one patient.

### Results According to Peak Thrombin Values

Because of the major differences in thrombin generation curves seen in patients, it was decided to divide patients into two groups based on whether they had a peak thrombin level ≥100 nM or <100 nM ( Table 3 ). The two groups consisted of five patients each, and the age and gender distribution was equal. Patients with a peak thrombin level ≥100 nM had lower absolute decreases in platelet counts than patients with a peak thrombin level <100 nM (36 [23–42] vs. 76 [46–132] × 10 ^9^ /L), and the same applied to relative decreases (30 [19–45] vs. 57 [45–62] %). Patients with ≥100 nM peak thrombin only demonstrated no thrombin generation in 0.75 IU/mL dalteparin samples, whereas the <100 nM peak thrombin group demonstrated no thrombin generation across all concentrations of dalteparin and argatroban. When analyzing samples without anticoagulant using INTEM, patients with peak thrombin ≥100 nM had slightly higher CT (181 [137–235] s vs. 272 [192–329] s) and higher MaxVel–t (193 [166–250] s vs. 324 [205–388] s) than in patients with values <100 nM. MaxVel and MCF did not differ between the two groups.

**Table 3:** Stratification of the study population ( *n* = 10) based on peak thrombin

Peak thrombin, nM ≥100 nM	268 Yes	227 Yes	261 Yes	105 Yes	111 Yes	12 No	50 No	54 No	64 No	6 No
Demography										
Age, years	55	73	79	55	67	48	68	74	74	85
Sex, M/F	M	M	F	F	F	F	F	M	M	M
Hemostatic parameters										
Platelets, ×10 ^9^ /L	97	99	86	41	58	45	65	96	57	83
Absolute platelet decrease, ×10 ^9^ /L	23	42	37	33	36	46	108	132	76	69
Relative platelet decrease, %	19.2	29.8	30.1	44.6	38.3	50.5	62.4	57.9	57.1	45.4
F1 + 2	++	+++	++	−	+	+++	++	++	−	+
TAT	−	+++	++	−	+	+++	−	++	+	−
Thrombin generation parameters										
*Lag time, min*										
Control	6.2	4.0	6.5	4.5	4.3	2.8	7.3	4.8	5.7	6.2
0.25 IU/mL DAL	5.3	4.3	6.3	4.0	3.3	3.0	flat	5.0	1.3	flat
0.75 IU/mL DAL	flat	6.0	flat	flat	flat	4.0	flat	6.3	flat	flat
0.25 µg/mL ARG	13.7	7.8	12.8	14.3	13.5	Flat	34.8	10.0	16.7	flat
0.50 µg/mL ARG	19.0	10.5	15.5	20.2	17.8	Flat	flat	11.6	11.6	flat
*Peak thrombin, nM*										
Control	268	227	261	105	111	12	50	54	64	6
0.25 IU/mL DAL	204	132	44	18	12	29	flat	62	11	flat
0.75 IU/mL DAL	flat	41	flat	flat	flat	9	flat	27	flat	flat
0.25 µg/mL ARG	174	137	251	24	41	flat	9	17	21	flat
0.50 µg/mL ARG	67	50	223	15	23	flat	flat	11	14	flat
*Time-to-peak, min*										
Control	8.7	6.5	9.8	7.7	8.3	7.7	11.7	8.5	10.3	16.0
0.25 IU/mL DAL	8.3	7.3	12.2	9.8	8.8	6.3	flat	8.5	11.3	flat
0.75 IU/mL DAL	flat	10.2	flat	flat	flat	8.8	flat	10.2	flat	flat
0.25 µg/mL ARG	16.3	10.0	15.5	23.7	20.7	flat	56.5	20.5	35.5	flat
0.50 µg/mL ARG	22.8	13.3	18.2	35.7	29.8	flat	flat	32.5	60.0	flat
*ETP, nM x min*										
Control	2,121	1,367	2,033	692	929	103	438	404	898	98
0.25 IU/mL DAL	1,795	856	562	254	112	221	flat	455	211	flat
0.75 IU/mL DAL	flat	373.7	flat	flat	flat	100	flat	236	flat	flat
0.25 µg/mL ARG	1,401	980	1,707	649	879	flat	432	559	799	flat
0.50 µg/mL ARG	1,172	823	1,466	674	853	flat	flat	559	382	flat
INTEM parameters										
*CT, s*										
Control	217	137	175	235	181	329	287	272	192	271
0.25 IU/mL DAL	241	163	208	212	212	351	396	294	216	289
0.75 IU/mL DAL	295	186	234	251	242	480	421	317	255	324
0.25 µg/mL ARG	282	185	241	273	249	603	442	337	268	353
0.50 µg/mL ARG	339	237	250	298	294	845	540	376	358	456
*MaxVel, mm/min*										
Control	19	15	20	13	17	6	8	11	16	13
0.25 IU/mL DAL	17	14	17	12	13	4	7	12	15	13
0.75 IU/mL DAL	15	15	16	11	13	4	7	12	14	12
0.25 µg/mL ARG	17	14	19	11	15	4	8	15	15	12
0.50 µg/mL ARG	14	14	20	11	11	2	9	14	12	11
*MaxVel-t, s*										
Control	250	166	190	250	193	388	324	328	205	322
0.25 IU/mL DAL	280	190	233	235	223	406	448	334	232	344
0.75 IU/mL DAL	345	229	262	290	262	610	545	376	280	373
0.25 µg/mL ARG	330	220	263	307	266	761	526	388	290	412
0.50 µg/mL ARG	395	297	280	358	325	1142	640	424	388	541
*MCF, mm*										
Control	64	59	61	48	58	45	56	57	61	59
0.25 IU/mL DAL	62	58	60	47	56	41	53	57	60	59
0.75 IU/mL DAL	62	61	58	52	59	38	56	62	63	62
0.25 µg/mL ARG	60	57	61	50	56	39	55	60	59	58
0.50 µg/mL ARG	61	62	63	53	54	28	59	62	56	57

Abbreviations: ARG, argatroban; CT, clotting time; DAL, dalteparin; ETP, endogenous thrombin potential; F1 + 2, prothrombin fragment 1 + 2; IU, international units; MaxVel, maximum velocity; MaxVel-t, time to maximum velocity; MCF, maximum clot firmness; TAT, thrombin-antithrombin complex.

Note: Data are shown as individual values for each patient. No thrombin generation is noted as “flat”. -, within reference range, +, above reference range, ++, two times above reference range; ++ + , three or more times above reference range.

## Discussion

The present study found that critically ill sepsis patients with new-onset thrombocytopenia had a decreased total amount of thrombin generated when spiking blood samples with dalteparin. Argatroban prolonged the initiation of thrombin generation and decreased the peak thrombin concentration but affected the total amount of thrombin generated to a lesser extent. Neither dalteparin nor argatroban affected the maximum velocity of clot formation or clot strength.

We designed an experimental study as a first step to improve the understanding of potential anticoagulant prophylaxes among a prevalent patient population. To our knowledge, no prior studies have investigated the impact of dalteparin and argatroban on the hemostasis among critically ill sepsis patients with new-onset thrombocytopenia. Therefore, it was difficult to contextualize our findings to the literature, as prior studies focused on anticoagulant effects in healthy individuals or other critically ill patient groups. 27 28 29 30 31 32 33 34 Furthermore, it was difficult to directly compare the *in vitro* effect between dalteparin and argatroban as these anticoagulants hold completely different pharmacological properties. 7

Regarding dalteparin and its effect on thrombin generation, Hacquard et al found that dalteparin dose-dependently inhibited thrombin generation in PPP samples from 12 healthy individuals, 27 similar to our findings. Interestingly, dalteparin did not seem to have major impact on the initiation phase of thrombin generation in our patient samples. This is also supported by Robert et al investigating the effect of enoxaparin in healthy individuals showing a potent inhibitory effect on ETP but no major affection of lag time. 28 A study using ROTEM in 16 healthy males demonstrated that dalteparin neither affected EXTEM-CT nor INTEM-CT when using therapeutic concentrations. 29 This is contrary to both previous finding 30 31 and our findings as these results suggest a significant effect on INTEM but not EXTEM parameters. The prolonged clot initiation induced by dalteparin in INTEM but not in thrombin generation could possibly be explained by dalteparin demonstrating no thrombin generation in most samples investigated, which could then result in a prolongation in lag time not being detected due to flattened curves.

When analyzing the effect of argatroban on thrombin generation in healthy and patient samples, we demonstrated an increase in lag time and time-to-peak and a decrease in peak thrombin, whereas ETP was not affected to the same extent as with dalteparin. These findings are in accordance with previous studies, 28 32 indicating that argatroban mainly inhibits the initiation phase and propagation phase of thrombin generation, while total thrombin generation is partially maintained. This finding was unexpected but might reflect low argatroban concentrations in the blood samples. However, argatroban may preserve the total amount of thrombin generated because of its ability to inhibit thrombin selectively and competitively, not completely shutting down the enzymatic capacity of thrombin generation. One must keep in mind that this is strictly hypothetical. In terms of ROTEM, increasing plasma concentrations of argatroban has been shown to significantly correlate with CT and MaxVel-t when using INTEM in both healthy individuals and critically ill patients suspected of heparin-induced thrombocytopenia. 33 34 This is consistent with the effect demonstrated by our results, where even low concentrations of argatroban caused major prolongations of clot initiation. Several other studies recommend the use of viscoelastic assays for monitoring of argatroban rather than aPTT. 33 34 35 This is also supported by our findings demonstrating a linear relationship between argatroban concentrations and CT using INTEM and EXTEM in healthy individuals. However, it should be emphasized that the monitoring of direct thrombin inhibitors using aPTT depends greatly on aPTT reagent manufacturers. 36 The results using aPTT ratios when adding argatroban are therefore not generalizable.

As reported in other studies, neither dalteparin nor argatroban affected clot strength in blood from critically ill patients in our study. 30 34 Although the patients were thrombocytopenic, they must have preserved platelet function and fibrin formation to maintain clot strength. Even when healthy and patient samples containing argatroban were evaluated by FIBTEM, where the platelet contribution to clot strength is eliminated, clot strength was maintained. Decreased total amount of thrombin generated measured by thrombin generation has been shown to be correlated with reduced clot strength. 37 38 Thus, there is a discrepancy in evaluating clot strength when using thrombin generation assays and ROTEM.

The majority of sepsis patients have an increased thrombin generation introducing a procoagulant state. 39 Hoppensteadt et al demonstrated that sepsis patients with suspected DIC, defined as low platelet counts and increased INR, had higher fibrin D-dimer, TAT levels, and F1 + 2 concentration than healthy controls. 40 Our study supports these findings as critically ill sepsis patients with new-onset thrombocytopenia had increased levels of D-dimer, TAT, and F1 + 2, reflecting increased *in vivo* thrombin generation and increased fibrin turnover. Despite the excessive *in vivo* thrombin generation, the *in vitro* thrombin generation potential in our patient samples varied 20-fold from the lowest to the highest amount of thrombin generated evaluated by ETP. It is unclear why some patients lose their ability to generate thrombin efficiently in an *in vitro* environment. By dividing patients in two groups according to peak thrombin, our study demonstrated that the decreases in platelet count within 24 hours prior to inclusion were lower in patients with peak thrombin levels ≥100 nM than in patients with levels <100 nM. Previous studies suggest that higher platelet counts increase the rate and peak of thrombin generation, 41 42 43 however, these studies focused on stable platelet counts and not new-onset thrombocytopenia, which demonstrates a more dynamically developed hemostatic abnormality. Platelets were depleted in plasma samples by centrifugation prior to analysis eliminating their contribution to thrombin generation. Thus, one could hypothesize that patients with less prominent decreases in platelet count might reflect a milder activation of the primary hemostasis and may not exhaust coagulation proteins through the secondary hemostasis *in vivo* , therefore maintaining the capacity to generate thrombin *in vitro* . In this context, it would have been interesting to investigate the difference between PPP and platelet-rich plasma (PRP) samples in the thrombin generation assay. Other studies have demonstrated that both PPP and PRP can be used for studies like ours. 44 45

## Strengths and Limitations

The strengths of the present study include the meticulous titration experiments performed in healthy individuals before moving on to spiking blood samples from patients. This enabled us to design the study in a clinically representative way. Next, we were able to establish a well-defined study population with early signs of coagulation abnormalities reflected by new-onset thrombocytopenia. Our study was limited by a relatively low number of patients and healthy individuals exploring novel anticoagulant effects on hemostatic laboratory assays.

## Conclusion

In conclusion, dalteparin had a major impact on the amount of thrombin generated, whereas argatroban mainly delayed clot initiation in blood samples from critically ill sepsis patients with new-onset thrombocytopenia. Neither anticoagulant affected clot strength. Further, the majority of patients had a high *in vivo* thrombin generation reflected by elevated levels of TAT and F1 + 2, while the hemostatic potential for *in vitro* thrombin generation differed substantially among patients.
