# Supplementary material for: In vitro Effect of Dalteparin and Argatroban on Hemostasis in Critically Ill Sepsis Patients with New-Onset Thrombocytopenia
Source: TH Open. 2023 Jan 30;7(1):e42–55. doi: 10.1055/a-2000-6576 (PMC9886503; doi:10.1055/a-2000-6576)
Supplement: Supplementary file 1 — Supplementary Material [file 10-1055-a-2000-6576-s22090042.pdf]

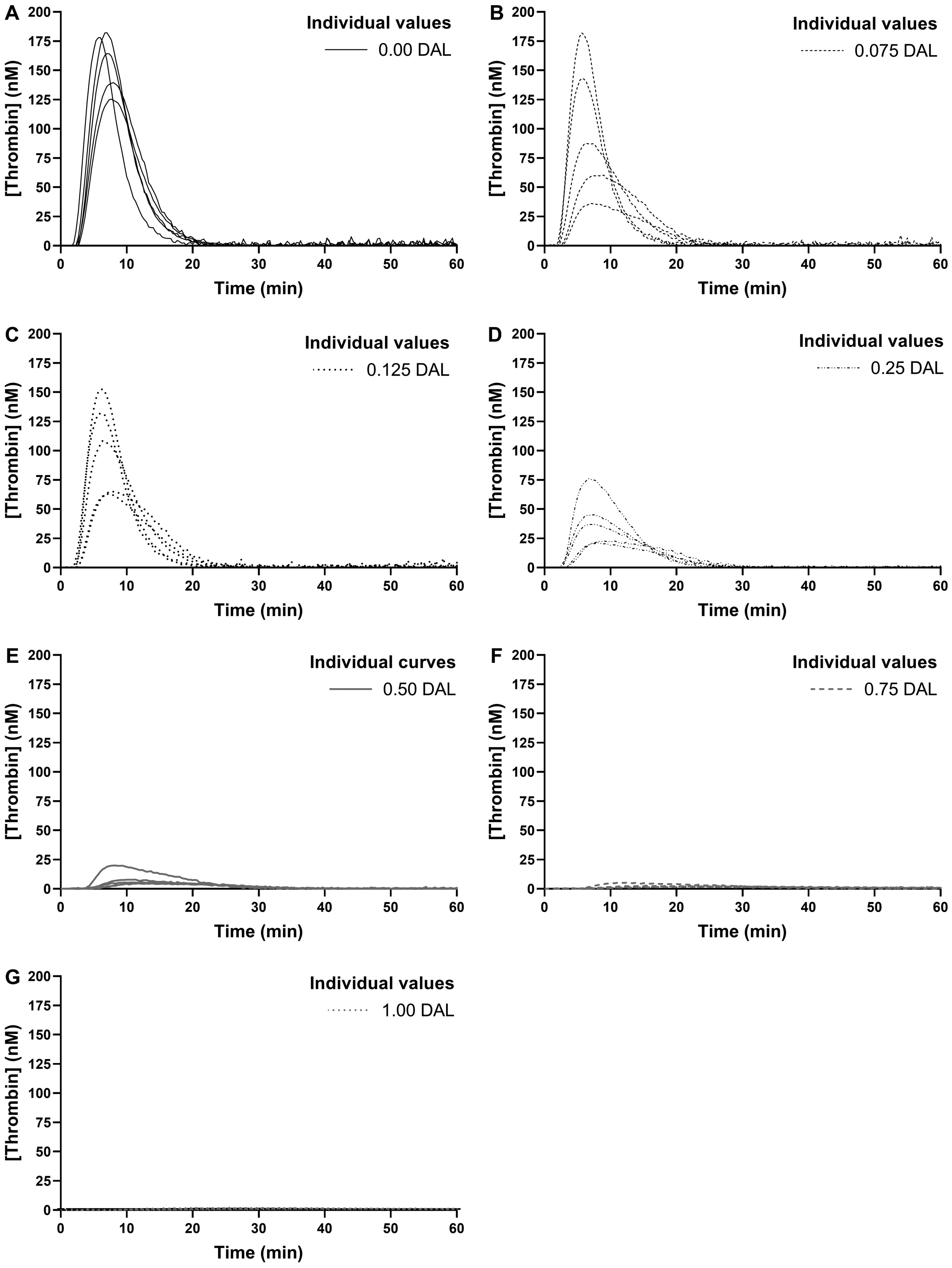

Supplementary Fig. S1. Effects of increasing concentrations of dalteparin (DAL, 0.00 to 1.00 IU/mL) on thrombin generation in platelet-poor plasma samples from healthy individuals ($n = 5$) triggered with 5 pM tissue factor and 4 µM phospholipids. Data are shown as individual curves.

 © 2023. The Author(s).

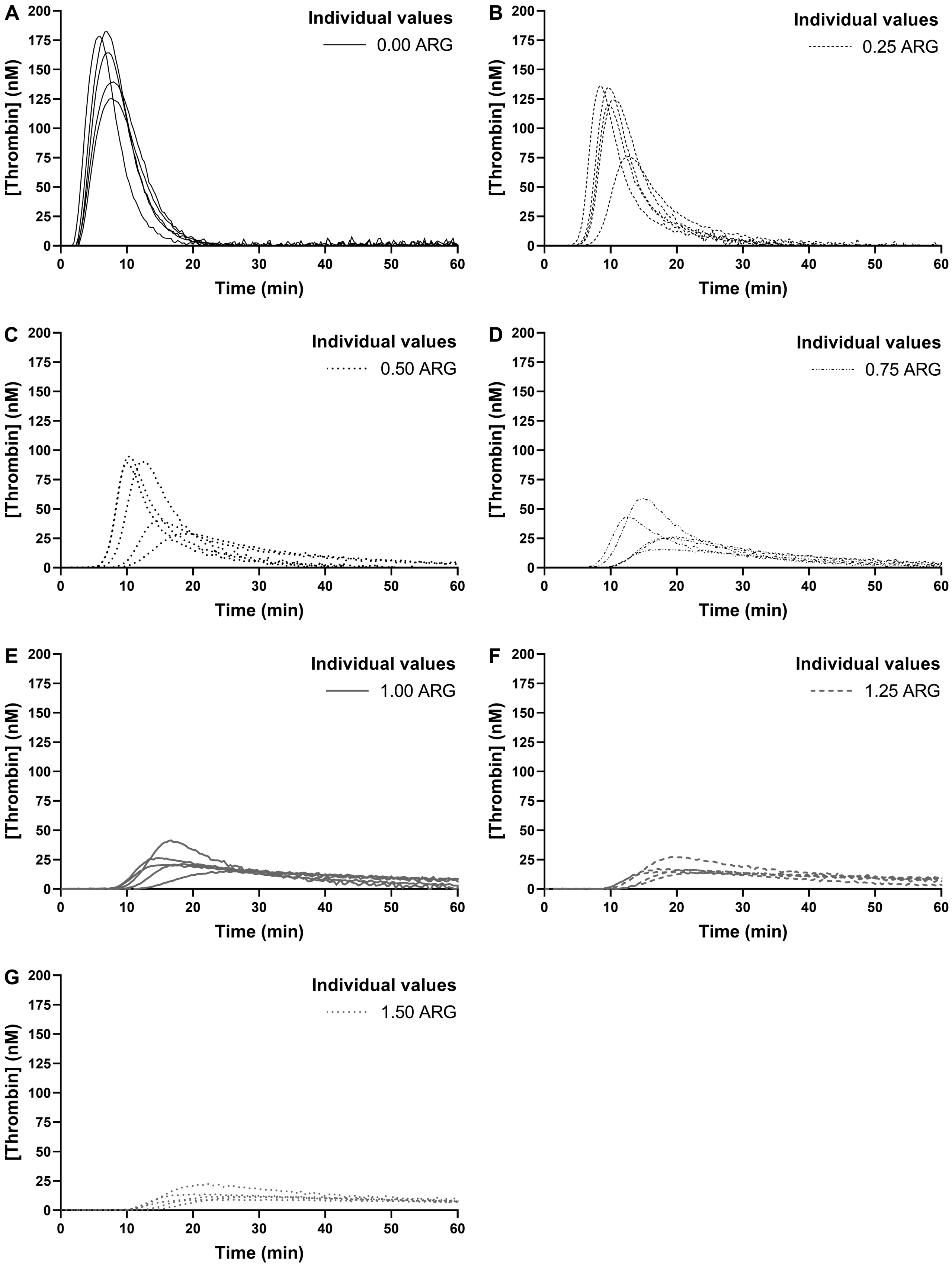

Supplementary Fig. S2. Effects of increasing concentrations of argatroban (ARG, 0.00 to 1.50 µg/mL) on thrombin generation in platelet-poor plasma samples from healthy individuals ($n = 5$) triggered with 5 pM tissue factor and 4 µM phospholipids. Data are shown as individual curves.

 © 2023. The Author(s).

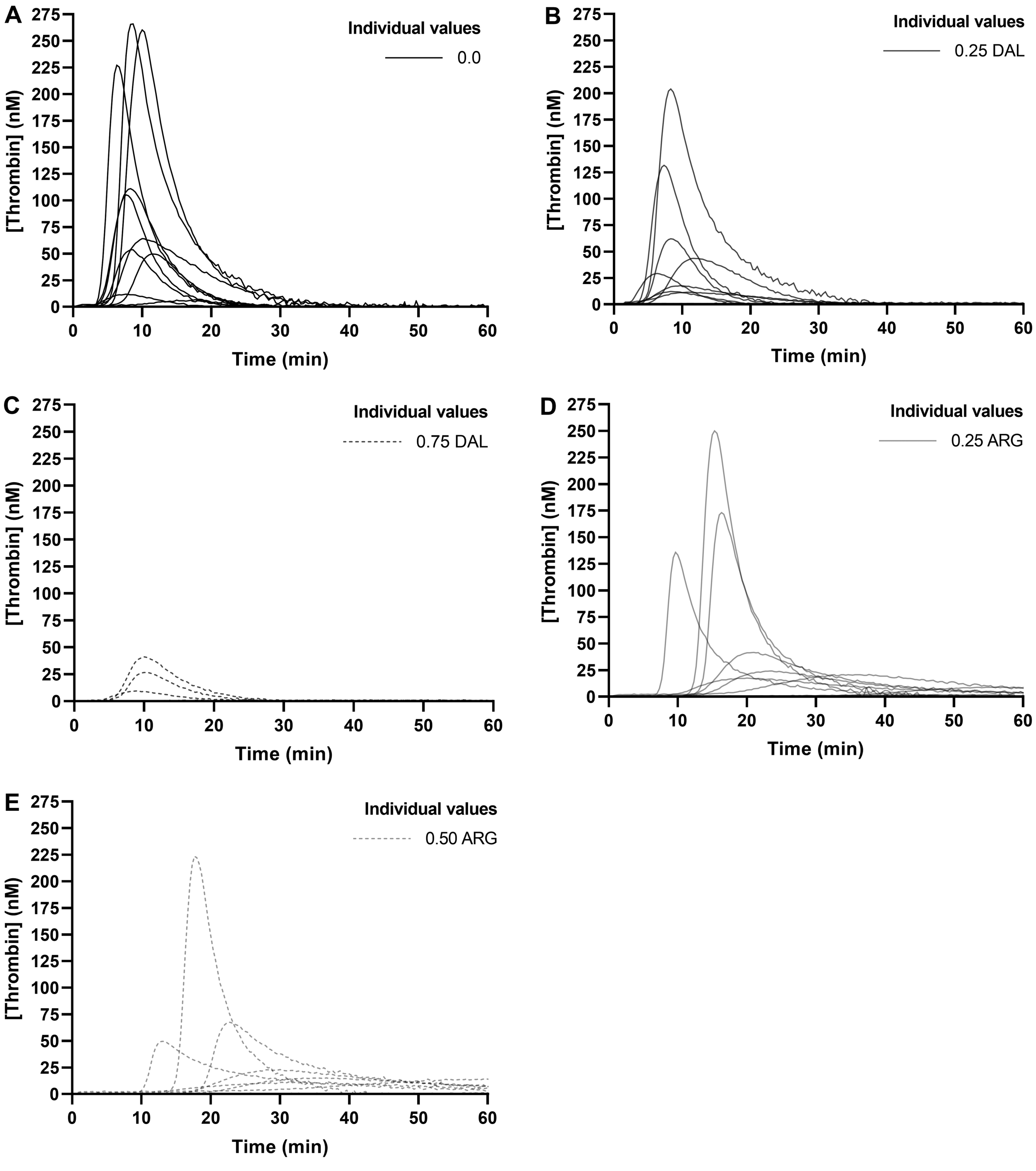

Supplementary Fig S3. Thrombin generation in platelet-poor plasma samples **(A)** without anticoagulant (0.00), **(B)** 0.25 IU/mL and **(C)** 0.75 IU/mL dalteparin samples, and **(D)** 0.25 µg/mL and **(E)** 0.50 µg/mL argatroban samples from critically ill sepsis patients with new-onset thrombocytopenia ($n = 10$) triggered with 5 pM tissue factor and 4 µM phospholipids. Data are shown as individual curves.

 © 2023. The Author(s).
